# Concurrent papular and dyshidrotic mycosis fungoides: A case report in a patient with skin of color

**DOI:** 10.1016/j.jdcr.2024.05.027

**Published:** 2024-06-13

**Authors:** Bryan Ma, Brian D. Rankin, Melissa Yanitski, Xiu Y. Jiang, Lesley Street, Jori Hardin

**Affiliations:** a Division of Dermatology, Department of Medicine, Cumming School of Medicine, University of Calgary, Calgary, Alberta, Canada; b Division of Hematology, Department of Medicine, Cumming School of Medicine, University of Calgary, Calgary, Alberta, Canada; c Department of Pathology and Laboratory Medicine, Cumming School of Medicine, University of Calgary, Calgary, Alberta, Canada

**Keywords:** cutaneous T-cell lymphoma, dyshidrotic mycosis fungoides, mycosis fungoides, papular mycosis fungoides, skin of color

## Introduction

Mycosis fungoides (MF) is the most common form of cutaneous T-cell lymphoma (CTCL).^1^ MF classically presents as variably scaly, atrophic, erythematous patches distributed on sun-protected sites with the potential to progress to plaques and tumors. Several clinical variants of MF have been described,^2^^,^^3^ but there have been limited reports of multiple clinical morphologic forms of MF appearing simultaneously, especially a combination of uncommon variants like papular or dyshidrotic MF. Furthermore, with the exception of hypopigmented MF, clinical variants in skin of color have been lacking.^4^ Therefore, we present a case report and literature review of a unique presentation of mycosis fungoides in a Black individual.

## Case report

A 57-year-old male who self-identified as Canadian with Ethiopian ancestry presented with a 2-year history of scattered intensely pruritic papules and vesicles that started on the flexor aspect of his forearms and progressively spread to include his posterior calves, thighs, and the palmar aspects of his hands bilaterally. He applied betamethasone valerate 0.1% cream and 0.1% ointment to affected areas intermittently to no effect.

**Fig 1:**
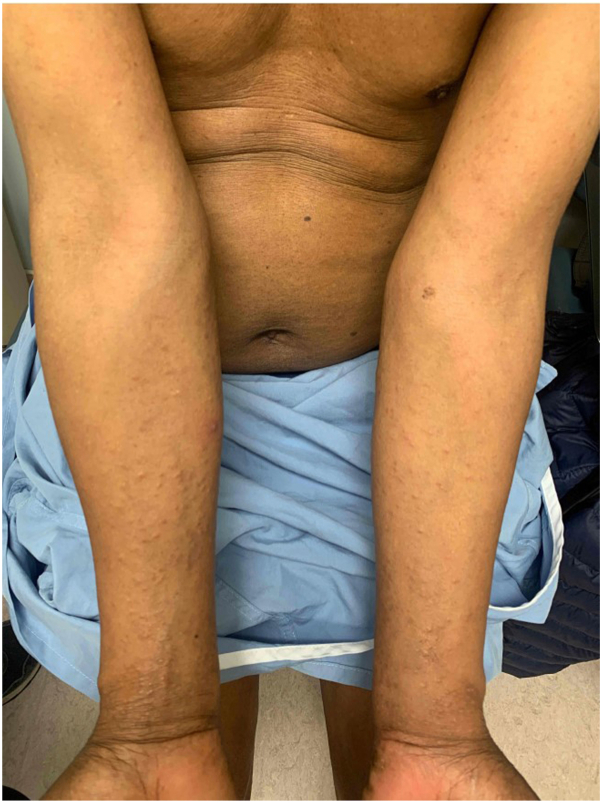
Papular mycosis fungoides of the bilateral ventral forearms.

**Fig 2:**
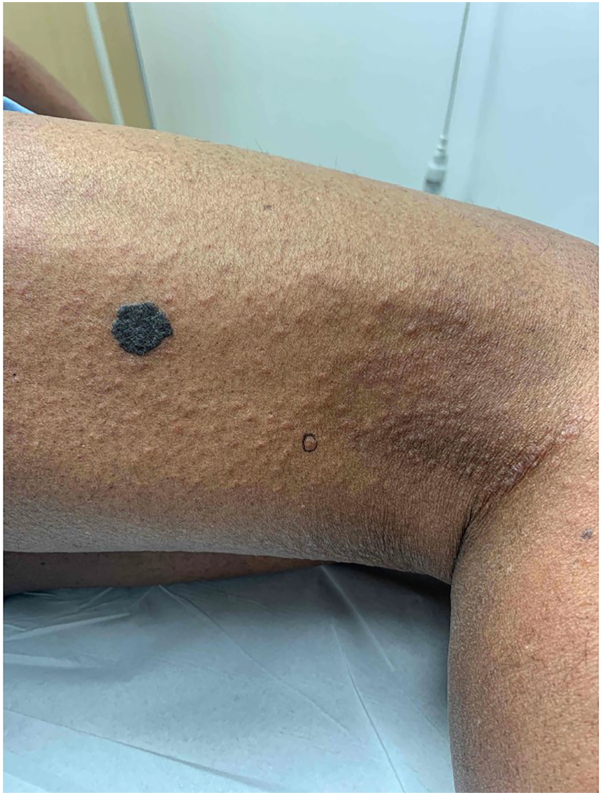
Papular mycosis fungoides of the right posterior thigh. Site of punch biopsy circled in *black ink*.

**Fig 3:**
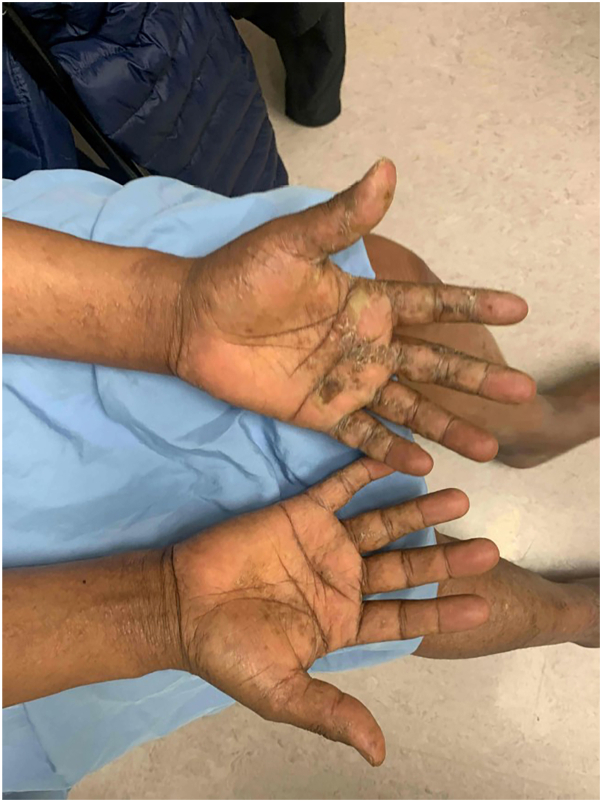
Vesicular and dyshidrotic mycosis fungoides of the right palmar surface.

On examination, there were erythematous lichenoid papules coalescing into plaques across the upper and lower extremities bilaterally. The palms of both hands had groups of clustered vesicles with a solitary tense bulla. Total affected body surface area was approximately 10%. There was no palpable lymphadenopathy (Fig 1, Fig 2, Fig 3).

Biopsy of the right lateral palmar surface from May 2022 showed spongiotic dermatitis with vesiculation and eosinophils. An initial biopsy of the left superior dorsal forearm showed spongiosis with a dense dermal lymphoeosinophilic infiltrate with abundant Langerhans cells. The infiltrate was composed of atypical monoclonal CD2/CD3/CD4/CD5 positive T cells. Repeat biopsy in 2023 of the right superior dorsal forearm showed an atypical monoclonal CD3/CD4 positive T-cell proliferation, an expansile superficial dermal and perivascular infiltrate of small and intermediate size lymphoid cells with convoluted nuclear contours, and cytologic atypia. The epidermis showed Pautrier’s microabscesses with subtle and focal dermo-epidermotropism affecting the basal layer. Immunohistochemistry showed a preponderance of CD2/CD3/CD4/CD5 positive T-cells with near total loss of CD7 expression.

**Fig 4:**
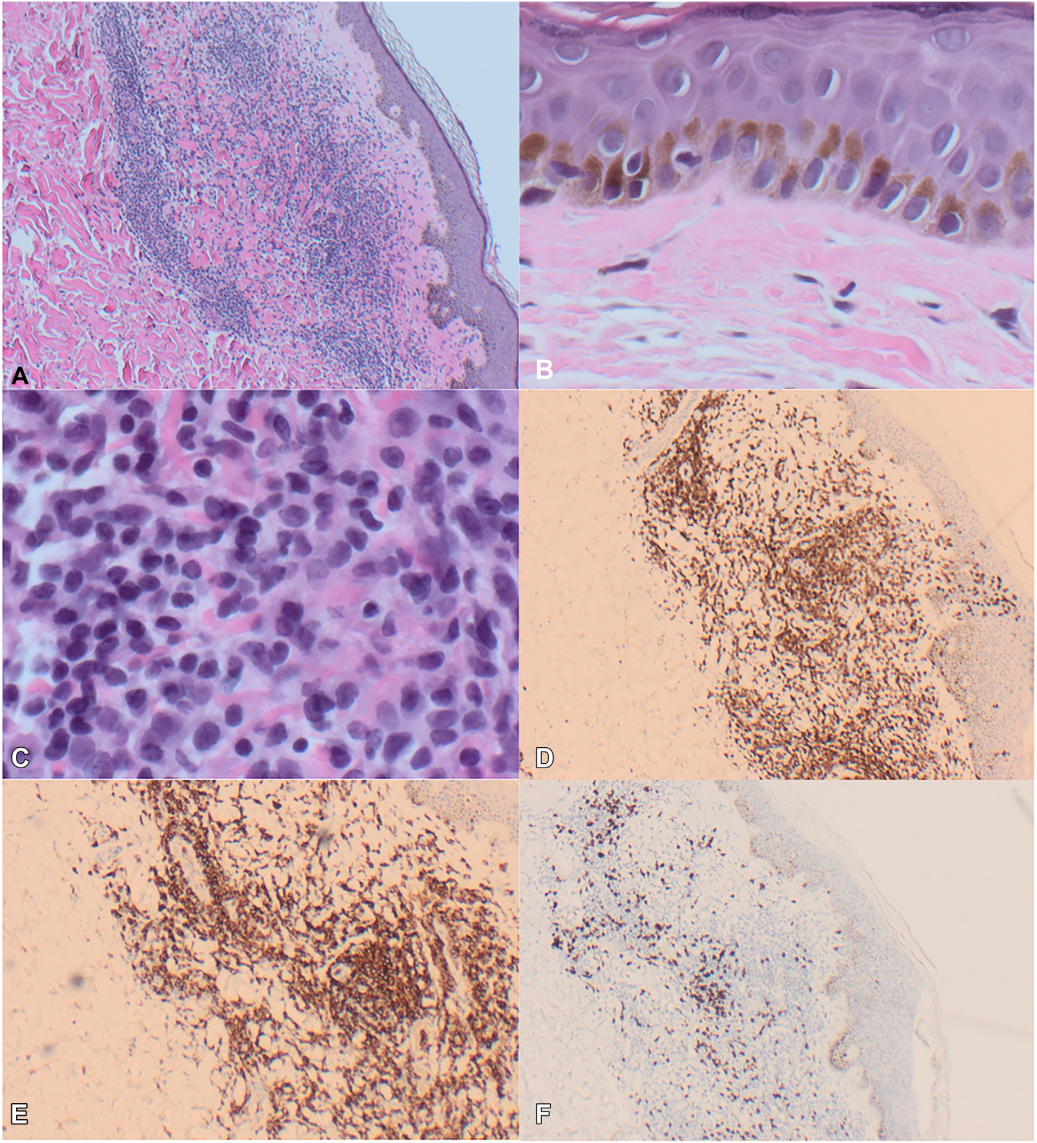
Representative pathology and immunohistochemistry slides. **A,** 4× magnification of the upper dermis showing a lymphocytic infiltrate (H&E); (**B**) 40× magnification showing rare lymphocyte exocytosis (H&E); (**C**) 40× magnification of small and medium atypical lymphoid cells (H&E); (**D**) CD3 positive T-cells (CD3 IHC); (**E**) CD4 positive T-cells (CD4 IHC); and (**F**) Minimal CD8 positive T-cells (CD8 IHC).

Given the clinical papular appearance, another punch biopsy was performed of the right posterior thigh. This showed rare exocytosis of lymphocytes into the epidermis and an infiltration of lymphoid cells in the upper dermis. Lymphocytes were small with slightly irregular nuclear contours and hyperchromatic nuclei. A monoclonal TCR beta and gamma gene rearrangement was identified, and this matched rearrangements found in previous biopsies extending back 1 year from the right palm and bilateral forearms, as well as in circulating lymphocytes in the blood. Immunohistochemistry showed CD2/CD3/CD4/CD5 positive T-cells that were negative for CD25 (Fig 4) with a CD4:CD8 ratio of approximately 8:1. Peripheral blood flow cytometry done twice showed atypical CD4+/CD7-/CD26- and CD4+/CD7-/CD26- clonal T-cell populations accounting for 18% and 24% of all lymphocytes (absolute count 540 and 720 cells per mm^3^), respectively. There was no follicular infiltration. Human T-lymphotropic virus 1 serology was negative. Serum lactate dehydrogenase was within normal limits.

Consequently, a final diagnosis of stage IB (T2N0M0B1b) papular and dyshidrotic mycosis fungoides was made. The patient declined escalation of treatment with phototherapy, systemic retinoids, and/or interferon. He improved dramatically with betamethasone valerate 0.1% ointment twice daily within 2 months and at 9 months of follow-up, his disease remains improved much with <1% affected BSA.

## Discussion

To the best of our knowledge, this is not only the first reported case of papular MF with concurrent dyshidrotic MF, but also the first description of a unique combination of MF morphologies in a patient with skin of color.

Papular MF was initially reported by Kodama et al^5^ in a case series of 6 patients. Since then, there have been less than 20 cases reported (Table I). Based on the reported cases, the median age of onset is 57 years with a slight male predominance (1.42:1). The most common presentation is discrete erythematous papules that are <1 cm in size distributed symmetrically across the trunk and extremities. Despite the unique clinical presentation, the histologic and immunohistochemical features of papular MF are generally similar to that of classic disease with epidermotropic, pleomorphic lymphocytic infiltrates. Numerous effective treatment regimens have been attempted including topical corticosteroids, phototherapy, psoralen with ultraviolet A, and extracorporeal photopheresis; most cases demonstrate a complete response, though relapsing and recurring lymphoma has been reported.^5^ Based on published case reports, this entity appears to have indolent clinical behavior with a 100% disease specific survival rate at a median of 36 months of follow-up. Only 2 cases were reported to have more than one clinical MF morphology. Therefore, this is only the third case of reported papular MF appearing simultaneously with another morphology, and the first case in combination with dyshidrotic MF, an even rarer morphology with only two other published reports of dyshidrotic disease in the literature (Table I).

**Table I:** Demographics, clinical presentation, and histopathology of previously reported cases of papular and dyshidrotic mycosis fungoides

Study and year	Patient number	Age and sex	Location	Concurrent or prior MF	Treatment	Best response	Follow-up duration
Kodama et al 2005	1	57 M	Trunk	No	PUVA	Complete response	18 mo
	2	58 F	Trunk and upper extremities	No	PUVA and ECP	Partial response	51 mo
	3	41 M	Lower extremities	Yes, patch stage disease detected months after diagnosis of papular MF with pathology confirming the diagnosis. Present concurrently.	PUVA	Complete response	132 mo
	4	59 M	Trunk and extremities	No	PUVA	Complete response	24 mo
	5	61 M	Back, flanks, and shoulders	No	PUVA	Complete response	72 mo
	6	57 F	Trunk, upper and lower extremities	Yes, classic patch MF appeared 3 y after diagnosis with pathology confirming diagnosis. Not present concurrently.	PUVA	Complete response	120 mo
Uddin et al 2007	7	31 F	Trunk, upper extremities	No	UVB	Responded to therapy, degree of improvement not reported	10 mo
Liu et al 2010	8	27 M	Chest, axillae, and left popliteal fossa	No	Topical high-potency corticosteroids daily with retinoids nightly, NB-UVB	Partial response	Not reported
Martorell-Calatayud et al 2010	9	50 F	Lateral trunk	No	Clobetasol dipropionate 0.1% ointment twice daily	Complete response	15 mo
	10	55 F	Trunk, buttocks, feet	No	PUVA	Complete response	20 mo
Neri et al 2011	11	47 F	Lower extremities	No	PUVA, spontaneous response	Complete response	60 mo
Noe et al 2013	12	83 F	Abdomen, trunk, proximal extremities	No	Topical corticosteroid, topical mechlorethamine, NB-UVB, resolution with ECP	Partial response	48 mo
	13	65 F	Chest, mid-lower back, abdomen	No	Topical mechlorethamine, PUVA, and then methotrexate	Partial response	36 mo
Brajon et al 2013	14	63 M	Trunk	No	Topical high-potency corticosteroid (clobetasol cream 0.05%), narrow-band ultraviolet B	Complete response	Not reported
Santamarina-Albertos et al 2014	15	55 M	Lower extremities	No	Topical high-potency corticosteroid (clobetasol cream 0.05%), PUVA	Complete response	18 mo
Balta et al 2015	16	33 M	Trunk, extremities	No	PUVA	Complete response	10 mo
Kimet al 2016	17	66 M	Trunk, extremities	No	Narrow-band ultraviolet B, topical high-potency corticosteroid	Stable disease	36 mo
Goto et al 2021	18	61 M	Trunk	No	Narrow-band ultraviolet B	Complete response	36 mo
Dyshidrotic MF							
Diehl et al 2011	1	73 F	Soles, ankles, and heels	No	NB-UVB, clobetasol 0.05% ointment twice daily	Partial response	15 mo
Juan-Carpena et al 2021	2	58 F	Palms and soles	No	Oral PUVA, topical 5% imiquimod, topical mechlorethamine, 16% MAL-PDT	Complete response	12 mo

Additionally, there have been no previously reported cases of either papular or dyshidrotic MF in a Black individual. CTCL is an important diagnosis for healthcare providers to consider in Black patients, especially since the typical morphology of MF may be more challenging to identify in darker skin tones, resulting in misdiagnosis for more common inflammatory diagnoses such as atopic dermatitis, psoriasis, or pityriasis alba.^6^ Currently, Black patients are underrepresented in MF case reports and randomized controlled trials despite CTCL disproportionately affecting Black patients with a higher incidence rate, earlier age of disease onset, and more aggressive malignancy at time of diagnosis compared to White patients.^7^, ^8^, ^9^ Large cancer database analyses have suggested that overall survival and disease-free survival in Black patients with MF are significantly poorer than those of White patients despite controlling for relevant disease, socioeconomic, and treatment factors.^10^ Therefore, further research is required to determine whether more uncommon and challenging to detect MF variants like papular and dyshidrotic MF may be associated with worse outcomes in Black patients.

In conclusion, we present a rare case of combination papular and dyshidrotic mycosis fungoides in a Black patient. CTCL is an important diagnosis for healthcare providers to consider in patients with skin of color, and dermatologists must be aware of the multiplicity of ways that MF and its clinical variants may present.

## Conflicts of interest

None disclosed.

## References

[1] Kaufman A.E., Patel K., Goyal K. (2020). Mycosis fungoides: developments in incidence, treatment and survival. J Eur Acad Dermatol Venereol.

[2] Ahn C.S., ALSayyah A., Sangüeza O.P. (2014). Mycosis fungoides: an updated review of clinicopathologic variants. Am J Dermatopathol.

[3] Geller S., Lebowitz E., Pulitzer M.P. (2020). Outcomes and prognostic factors in African American and black patients with mycosis fungoides/Sézary syndrome: retrospective analysis of 157 patients from a referral cancer center. J Am Acad Dermatol.

[4] Miyashiro D., Sanches J.A. (2023). Mycosis fungoides and Sézary syndrome: clinical presentation, diagnosis, staging, and therapeutic management. Front Oncol.

[5] Kodama K., Fink-Puches R., Massone C., Kerl H., Cerroni L. (2005). Papular mycosis fungoides: a new clinical variant of early mycosis fungoides. J Am Acad Dermatol.

[6] Flaum-Dunoyer P., Noor S.J., Myskowski P.L. (2023). Cutaneous lymphomas in African American/Black patients: pitfalls and presentations. Int J Dermatol.

[7] Wilson L.D., Hinds G.A., Yu J.B. (2012). Age, race, sex, stage, and incidence of cutaneous lymphoma. Clin Lymphoma Myeloma Leuk.

[8] Johnson W.T., Kartan S., Sokol K., Nikbakht N., Porcu P. (2021). Clinical characteristics and outcomes of black patients with mycosis fungoides and Sézary syndrome: a subgroup analysis of the phase III MAVORIC trial. Leuk Lymphoma.

[9] Allen P.B., Goyal S., Niyogusaba T. (2022). Clinical presentation and outcome differences between Black patients and patients of other races and ethnicities with mycosis fungoides and Sézary syndrome. JAMA Dermatol.

[10] Allen P.B., Goyal S., Iyer S.P. (2022). Differences in progression and survival among black patients with mycosis fungoides and Sézary syndrome: a multicenter retrospective analysis at large urban medical centers. Blood.

